# Prognostic factors of patients with Gliomas – an analysis on 335 patients with Glioblastoma and other forms of Gliomas

**DOI:** 10.1186/s12885-019-6511-6

**Published:** 2020-01-15

**Authors:** Jianfeng Liang, Xiaomin Lv, Changyu Lu, Xun Ye, Xiaolin Chen, Jia Fu, Chenghua Luo, Yuanli Zhao

**Affiliations:** 1 Department of Neurosurgery, Peking University International Hospital, No.1 Science Park Road, ZGC Life Science Park, Beijing, 102206 China; 2 Department of Neurology, The First Hospital of Jilin University, Changchun, 130021 Jilin Province, China; 3 Department of Neurosurgery, Beijing Tiantan Hospital, Capital Medical University, Beijing, 100050 China; 4 Department of Retroperitoneal Tumors Surgery, Peking University International Hospital, No.1 Science Park Road, ZGC Life Science Park, Beijing, 102206 China

**Keywords:** Gliomas, Glioblastoma, Clinical characteristics, Survival, Prognostic factors

## Abstract

**Background:**

The prognosis of glioma is poor, despite recent advances in diagnosis and treatment of the disease. It is important to investigate the clinical characteristics and prognostic factors of glioma so as to provide basis for treatment and management of patients.

**Method:**

A total of 335 patients with glioma were included in this study. These patients were admitted to the medical center between November 2015 and December 2018. The clinical data, including demographic data, tumor characteristics, treatment strategy, expression pattern of tumor markers, and survival data, were retrospectively reviewed. Survival data were analyzed using Kaplan-Meier curves with log-rank test, while multivariate analysis Cox regression model was used to investigate risk factors for mortality.

**Results:**

In this patient cohort, glioblastoma (40%), diffuse glioma (14.6%) and oligodendroglioma (9.6%) were the most common pathological types. The expression of Ki-67 was associated with several clinicopathological parameters (e.g. tumor type, grade, and number of lesions). In addition, Ki-67 correlated with the mortality within the first year of the post-treatment follow-up (*P* <  0.001). Kaplan-Maier analysis revealed that older patients (≥ 45 years) displayed worse prognosis than those aged under 45 years (*P* = 0.038). Dismal prognosis was also associated with clinical parameters, including high tumor grade, multiple lesions, and Karnofsky performance score (KPS). Multivariate analysis showed that low KPS (< 85) increased the risk of mortality by 2.3 folds with a 95% CI of 1.141 to 4.776 (*P* = 0.020). Low tumor grade (grade 1–2) oppositely reduced the mortality risk by 0.22 folds (95% CI, 0.065 to 0.763, *P* = 0.0168).

**Conclusion:**

KPS and tumor grade were independent prognostic factors in patients with gliomas.

## Background

There are more than 100,000 cases of central nervous system (CNS) cancer diagnosed each year worldwide [1], and gliomas represent 40% of all brain tumors [2]. There are different types of gliomas, namely astrocytoma, oligodendroglioma, glioblastoma (GBM), and diffuse glioma. Among which, GBM is the most common brain neoplasms [3]. Studies showed that the disease was slightly male predominance (male to female ratio of approximately 1.4:1) [4, 5]. The clinical presentation of a patient with GBM can vary greatly depending on the stage and location of the tumor; some symptoms include headache, seizure, and progressive neurologic deficits, in which seizure is a symptom observed in as many as 25% of patients [6, 7].

Current standard therapy for gliomas includes maximal safe surgical resection, radiation, and chemotherapy with temozolomide [8]. Although there have been recent advances in diagnosis and treatment of the malignancy, the prognosis of gliomas is still poor, especially for those patients with malignant and invasive gliomas. The highly invasive nature prevents complete resection of the tumor, causing significant neurologic morbidity and mortality. For example, the median survival of patients with GBM was only 15 months and a median progression-free survival was 6.2 to 7.5 months [3, 5]. To improve the quality of life and survival time of patients, it is important to investigate prognostic factors of the disease, so the high risk patient group can be identified and treated with an aggressive regime. Some clinical-pathological parameters have been proposed. For example, Ki-67 was found as a reliable indicator of tumor cell proliferation [9], but its value in predicting prognosis remains controversial.

In this study, we investigated clinical manifestations and prognostic factors of patients with gliomas through a retrospective analysis of clinical characteristics and follow-up data of 335 patients. The data might provide insights for improving the treatment and management of patients with different types of gliomas.

## Patients and methods

### Study population

The present study was a retrospective study on 335 patients with gliomas. These patients were admitted to our hospital for medical treatment between November 2015 and December 2018. Eligible patients included those with their gliomas pathologically confirmed. Patients were excluded if they had other brain tumors or systemic diseases. The study was reviewed and approved by the Ethics Committee and Institutional Review Board of our hospital. Informed consent was obtained from each of the participants.

### Data collection

Clinical data were retrieved for statistical analysis from patient’s medical records. The data included patient’s demographic data, tumor characteristics (i.e. lesion sites, pathological classification, grade, etc.), treatment approaches (i.e. surgical resection, radiotherapy, and chemotherapy), tumor markers expression (i.e. Ki-67, GFAP, p53, etc.), and survival time after treatment.

### Statistical analysis

Retrieved data were analyzed using statistical software SAS version 9.3. Normally distributed continuous variables were analyzed using Student’s t-test, with the ones not normally distributed be examined using Wilcoxon two sample test. Survival data were studied using Kaplan-Meier curve with log-rank test, with the differences between survival curves evaluated using Tukey-Kramer corrected *P* values. Risk factors for mortality after treatment were identified by multivariate analysis using Cox regression model. Significant differences were indicated by *P* value < 0.05.

## Results

### Baseline characteristics of the enrolled patients

A total of 335 patients with glioblastoma were enrolled in this study (Table 1). There were 190 males and 145 females, with their ages ranging from 1 to 86 years. Epilepsy was complained in 97 patients (29%). Glioblastoma (40%), diffuse glioma (14.6%) and oligodendroglioma (9.6%) were the three most common pathological types, and for tumor grading, 46.7% of the cases were classified as grade 4.

**Table 1:** Demographic data, tumor characteristics and treatment strategies of patients

Clinicopathological parameters		Frequency(%)
Gender	Male	190 (56.72)
	Female	145 (43.28)
Epilepsy	No	238 (71.04)
	Yes	97 (28.96)
Location of lesion	Parietal and/or junction	35 (10.45)
	Forehead	89 (26.57)
	Fronto-temporal	18 (5.37)
	Brainstem	12 (3.58)
	Cerebellum	10 (2.99)
	Right temporal	77 (22.99)
	Left temporal	21 (6.27)
	Other	73 (21.79)
Pathological type	Malignant astrocytoma	21 (6.27)
	Malignant oligodendroglioma	16 (4.78)
	Glioblastoma	134 (40.00)
	Diffuse glioma	49 (14.63)
	Oligodendroglioma	32 (9.55)
	Astrocytoma	23 (6.87)
	Other	23 (6.87)
	Unknown	37 (11.04)
Tumor grade	1	10 (3.21)
	2	87 (27.88)
	3	60 (19.23)
	4	155 (49.68)
Lesion number	Single	303 (90.45)
	Multiple	32 (9.55)
Surgical treatment	No	56 (16.72)
	Yes	279 (83.28)
Surgical approach	Subtotal resection	67 (24.01)
	Gross resection	205 (73.48)
	Unknown	7 (2.51)
Radiotherapy	No	51 (15.22)
	Yes	115 (34.33)
	Unknown	169 (50.45)
Chemotherapy	No	52 (15.52)
	Yes	116 (34.63)
	Unknown	167 (49.85)

Most patients (83.3%) were treated by surgery, of which subtotal resection and gross total resection constituted 24 and 73.5% of the cases, respectively. These surgical approaches were associated with neither patients’ survival outcomes nor any clinicopathological parameters. About one-third of the patients received radiotherapy (34.3%) and chemotherapy (34.6%). Survival outcomes after treatment were followed up. Up to the time point when data were retrieved for analysis, the survival and mortality rate was 51.8 and 20%, respectively (i.e. 28.2% of the cases were not followed).

### Clinical correlation of Ki-67 level

The associations of Ki-67 with clinicopathological parameters were studied in 232 of 335 enrolled patients, who had clinical record of Ki-67 level (Table 2). Ki-67 level was associated with patients’ age (*P* <  0.001). The level also correlated the occurrence of epilepsy (*P* = 0.007). For pathological parameters, Ki-67 was associated with tumor type (*P* <  0.001), grade (*P* <  0.001), and number of lesions (single vs multiple) (*P* = 0.023). Ki-67 also correlated with Karnofsky performance score (KPS; *P* = 0.017) and several markers like neuronal nuclei (NeuN; *P* = 0.003), epithelial membrane antigen (EMA; *P* <  0.001), p53 (*P* <  0.001) and NF (*P* = 0.023).

**Table 2:** Correlation of Ki-67 with clinicopathological parameters in gliomas

Clinicopathological parameters		Ki-67 level		*P* value
		Low (< 20)	High (≥ 20)	
Age	< 45	77	35	< 0.001
	≥ 45	39	81	
Epilepsy	No	70	89	0.007
	Yes	46	27	
Pathological type	Malignant astrocytoma	9	4	< 0.001
	Malignant oligodendroglioma	2	11	
	Glioblastoma	16	82	
	Diffuse glioma	30	6	
	Oligodendroglioma	25	0	
	Astrocytoma	16	1	
	Other	18	12	
Tumor grade	1–2	71	1	< 0.001
	3–4	42	113	
Lesion number	Single	112	103	0.023
	Multiple	4	13	
KPS	< 85	58	76	0.017
	≥ 85	58	40	
NeuN	Positive	21	7	0.003
	Negative	86	106	
EMA	Positive	7	32	< 0.001
	Negative	95	76	
P53	Positive	58	97	< 0.001
	Negative	38	12	
NF	Positive	49	43	0.023
	Negative	10	23	

**Fig. 1:**
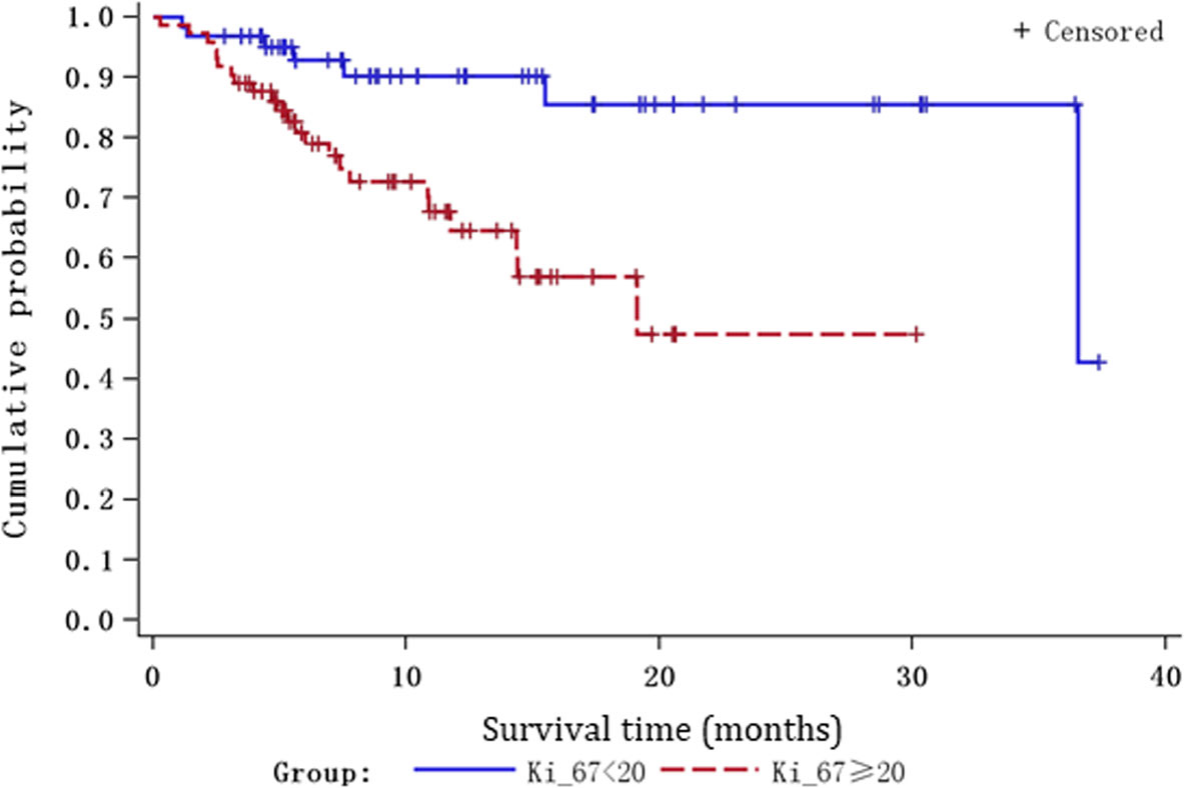
Kaplan-Meier analysis showing the significant difference in survival between glioma patients with low and high Ki-67 expression

Ki-67 was found not significantly associated with whether and how the surgery was done. Notably, Ki-67 correlated strongly with the mortality within the first year of the post-treatment follow-up (*P* <  0.001). In addition, patients with elevated Ki-67 (i.e. ≥ 20) showed shorter survival than those with low Ki-67 (i.e. < 20) within a post-treatment period of 40 months (*P* = 0.002) (Fig. 1).

### Clinicopathological parameters associated with the first-year survival

The factors affecting the first year survival of patients after treatment were examined (Table 3). Univariate analysis suggested that tumor pathological type and grade were only marginally associated (*P* = 0.05). The first-year survival also correlated with KPS (*P* <  0.001) and different markers including Ki-67 (*P* = 0.004), and Syn (*P* = 0.048). Multivariate analysis using Cox regression model was also performed to identify the risk factor of mortality within the first year post-treatment. It was shown that low KPS (i.e. < 85) was the only factor that substantially elevated the risk (OR, 5.965; 95% CI, 1.996 to 17.822; *P* = 0.001).

**Table 3:** Clinicopathological parameters associated with the first-year survival after treatment

Clinicopathological parameters		Survival	Mortality	*P* value
KPS	< 85	24	26	< 0.001
	≥ 85	45	6	
Ki-67	< 20	26	5	0.004
	≥ 20	21	20	
Syn	Positive	33	17	0.048
	Negative	9	0	

### Clinicopathological parameters associated with the overall survival

**Fig. 2:**
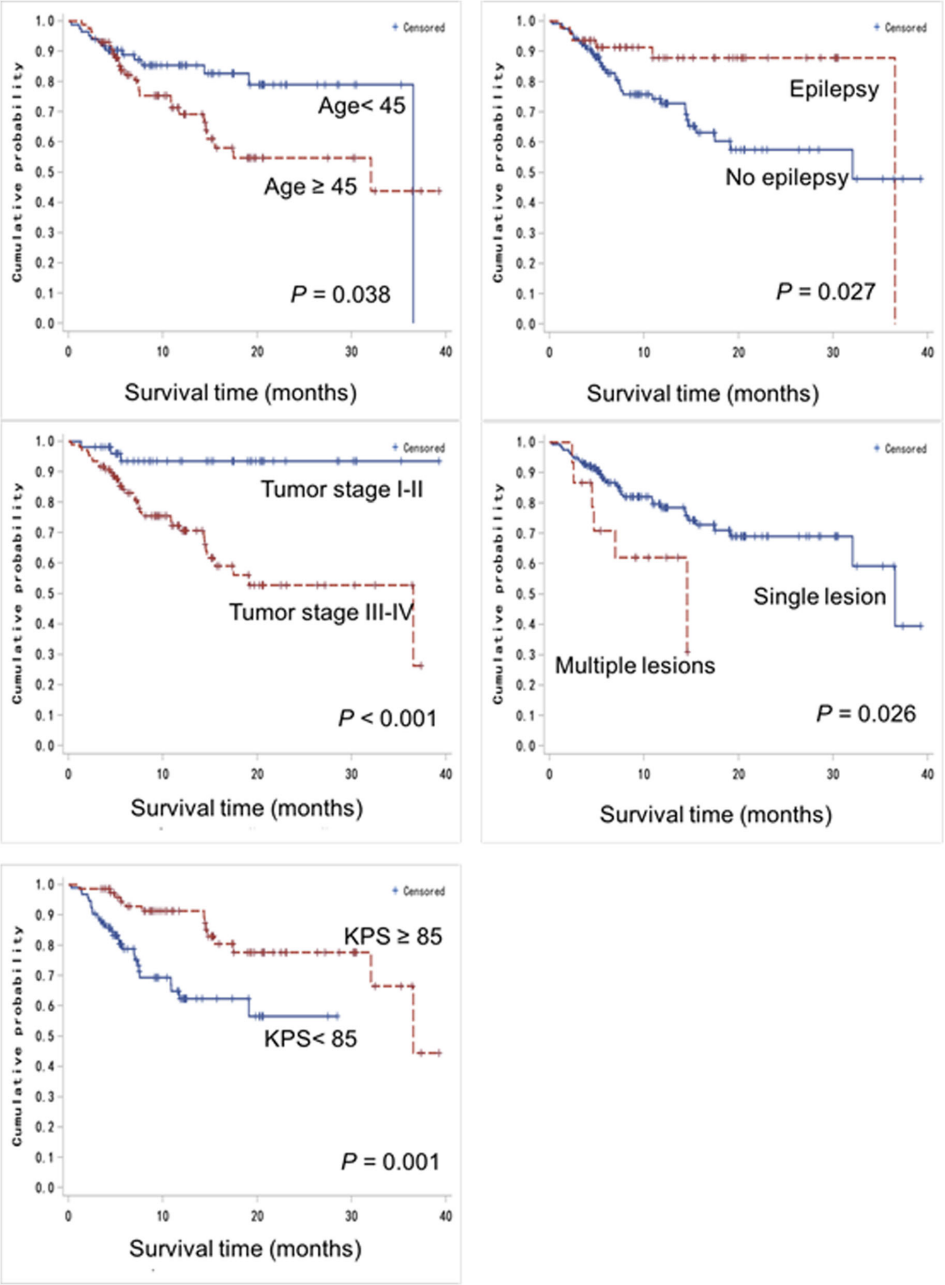
Significant association of clinical parameters with the overall survival of glioma patients as examined by Kaplan-Meier analysis

Kaplan-Maier analysis with log rank test revealed multiple clinicopathological parameters associated with patients’ overall survival (Fig. 2). Older patients (i.e. ≥ 45 years) displayed worse prognosis than those aged under 45 years (*P* = 0.038). Patients with no epilepsy also showed a poorer prognosis compared to their counterparts with epilepsy (*P* = 0.027). Dismal prognosis was also associated with certain tumor pathology like high tumor grade (i.e. grade 3–4) (*P* <  0.001) and multiple lesions (*P* = 0.026). Patients with KPS <  85 also presented less favorable survival outcomes compared to those with KPS ≥ 85 (*P* = 0.001).

Low KPS (i.e. < 85) was identified by multivariate analysis as a factor to increase the risk of mortality by 2.3 folds with a 95% CI of 1.141 to 4.776 (*P* = 0.020). Low tumor grade (i.e. grade 1–2) oppositely reduced the mortality risk by 0.22 folds (95% CI, 0.065 to 0.763, *P* = 0.0168).

## Discussion

Surgical resection and postoperative radiotherapy and chemotherapy can significantly delay tumor progression, however, the recurrence rate is unacceptably high, making the overall cure rate of gliomas low. With a hope to improve the treatment outcome of patients, there has long been a search for clinical parameters of prognostic values in the clinical decision of treatment strategy. Early studies have suggested many factors affecting the prognosis of glioma patients, however, most have remained elusive in their effects [10, 11]. Other clinical parameters like tumor grade, age of onset, surgical approaches, and the use of postoperative adjuvant therapy are also shown to be associated with the prognosis of patients [12, 13]. Over the last decade, with the advances in genomic and proteomic profiling, many molecular markers have emerged as prognostic indicators for glioma. A four-microRNA signature was shown able to identify patients with lower-grade gliomas under high risk of mortality [14]. A low serum level of microRNA-376 was identified as an independent factor predicting poor outcome of glioma patients [15]. A mutation of BRAF, V600E, was associated with an improved overall survival among glioma patients [16]. However, despite the usefulness of these molecular markers in predicting survival in their respective defined single cohorts, the prognostic power of these markers across multiple patient populations has yet to be further validated. It has remained important to examine the clinical parameters associated with the treatment outcome of glioma patients.

The present study examined the clinical associations of different clinicopathological parameters in 335 patients with glioma. Our analysis clearly suggested that old age, high tumor grade, multiple lesions, and low KPS are associated with the poor survival of the patients. Multivariate analysis further indicated that low KPS and low tumor grade can significantly elevate and reduce, respectively, the risk of mortality of patients. These findings are clinically relevant. The cohort size of the present study is relatively large when compared to those of the published, covering patients with different types of gliomas and those treated with different therapies. In addition, among the different types of gliomas found in our cohort, 40% of them were glioblastoma. This resembles most clinical situations in which glioblastoma is the most common types of gliomas worldwide.

Our analysis suggested that low KPS is an independent risk factor for mortality within the first year after treatment and in long-term survival of glioma patients. This finding is in line with many studies showing KPS is a promising prognostic indicator in patients with glioma. For example, patients with KPS > 70 were shown to survive longer than their counterparts with KPS < 70 [17]. The combined use of KPS with MGMT promoter methylation and patient age in a recursive partitioning analysis modal can also accurately predict the prognosis of patients with glioblastomas [18]. Our analysis also identified low tumor grade as a predictor of good survival. Indeed, tumor grades and KPS score are known clinical parameters already in practice. However, the present study defined a cutoff value of KPS that would be useful in Chinese patients with gliomas. Whether the combined use of KPS and tumor grade would further improve the prediction has yet to be fully examined.

## Conclusion

In WHO 2016 classification of brain tumors, molecular sub-grading has been established and is presently in routine clinical use. To provide more useful insights into the refinement of management strategy for patients with gliomas, the present study was aimed identify several clinical parameters associated with the prognosis of patients. The findings nevertheless require further validation in independent cohorts of glioma patients. In our study cohort, although patients received different treatments like radiotherapy, chemotherapy or combined treatment, the association of these treatments with patient’s prognosis has remained to be established. The prognostic value of clinical parameters combined with molecular markers has also yet to be examined. Furthermore, in vitro and in vivo experiments will be done to depict the clinical significance of certain molecular parameters in glioma.

## Data Availability

The datasets generated and analyzed during the current study are available from the corresponding author on reasonable request.

## Abbreviations

GBM: Oligodendroglioma, glioblastoma
KPS: Karnofsky performance score
